# Research on dense object detection methods in congested environments of urban streets and roads based on DCYOLO

**DOI:** 10.1038/s41598-024-51868-0

**Published:** 2024-01-11

**Authors:** Shuhai Jiang, Bowen Luo, Haoyue Jiang, Zhongkai Zhou, Shangjie Sun

**Affiliations:** 1 School of Mechanical and Electronic Engineering, Nanjing Forestry University, Nanjing, 210037 Jiangsu China; 2 Institute of Intelligent Control and Robotics (IICR), Nanjing Forestry University, Nanjing, 210037 Jiangsu China; 3 College of Letters and Science, University of Wisconsin-Madison, Madison, WI 53706 USA

**Keywords:** Engineering, Electrical and electronic engineering, Mechanical engineering

## Abstract

The urban street is a congested environment that contains a large number of occluded and size-differentiated objects. Aiming at the problems of the loss of the target to be detected and low detection accuracy resulting from this situation, a newly improved algorithm, based on YOLOv4, DCYOLO is proposed. Firstly, a Difference sensitive network (DSN) is introduced to extract the edge features of objects from the original image. Then, assign the edge features back to increase the edge intensity of the object in the original image and ultimately improve the detection performance. Secondly, the feature fusion module (CFFB) based on context information is introduced to realize the cross-scale fusion of shallow fine-grained features and deep-level features, to strengthen the cross-scale semantic information fusion of feature maps and eventually improve the performance of object detection. At last, in the network prediction part, the SIOU loss function replaces the original CIOU loss function to improve the convergence speed and accuracy of object detection. The experiments based on MS COCO 2017 and self-made datasets show that, compared with the YOLOv4, the detection accuracy of DCYOLO models is greatly improved with an increase of 9.1 percentage points in AP and 10.4 percentage points in AP_s_. Compared with YOLOv5x and Faster R-CNN, DCYOLO shows higher accuracy and better detection performance. The experiment result proves that the DCYOLO algorithm can adapt to the dense object detection requirements in the congested environment of urban streets.

## Introduction

Accompanied by the rapid development of artificial intelligence, intelligent transportation technology has received focused attention from a wide range of researchers. Therefore, as an important part of intelligent transportation, road object detection has become a key issue in current research. The main task of road target detection is to provide necessary information for intelligent transportation systems by identifying and locating various traffic signs, pedestrians, vehicles, and other obstacles in the captured images. Therefore, accurate detection of these targets is crucial to ensure the safe passage of vehicles and pedestrians.

Traditional feature classifiers such as Haar^1^, HOG^2^, and SIFT^3^ require manual feature selection and much debugging, making them low in accuracy and robustness and unable to adapt to complex road environments. Therefore, deep learning-based road object detectors that can learn features automatically are emerging and perform well. Object detector is usually categorized into two kinds, i.e., one-stage object detector and two-stage object detector. The two-stage object detector, such as R-CNN^4^, fast R-CNN^5^ and faster R-CNN^6^ are region-based. Due to the existence of the region proposal, a two-stage object detector performs well in detection accuracy but has a low detection speed. This is very unsuitable for complex and changing traffic scenarios However, a one-stage object detector skips the stage of region proposal and accomplishes classification and localization of the object directly in the image. Therefore, compared to a two-stage object detector, a one-stage model has a faster speed but lower detection accuracy. The most representative one-stage models are YOLO, SSD, and RetinaNet.

Although, after several generations of improvements, the one-stage model has an accuracy that matches or even exceeds that of the two-stage model but does not perform well in an urban street environment with dense objects. Urban street is an environment with a numerous but relatively fixed variety of objects that contains many occluded as well as size-differentiated targets. This situation exposes two shortcomings of deep learning-based target detection. One is that, in the feature extraction stage, the details of shallow features will be lost as the abstraction of the extracted features increases. This happens especially with high sampling multiple. The inevitable loss of information, in most situations, has little effect on the detection of conspicuous targets. However, it is very likely to decrease the detection accuracy of small-sized and occluded objects. The other problem is the always limited receptive field. A larger receptive field means less effective feature extraction for small-sized targets. Conversely, a smaller receptive field means that larger targets cannot be observed completely. This contradiction leads to poor detection performance on targets with large size differences. To deal with the above two problems, a new object detection algorithm DCYLOLO (Difference Sensitive and Context-based YOLO) is proposed based on the YOLOv4^7^ algorithm, which is innovative in the following three aspects.

1. *Introduction of Difference Sensitive Network (DSN)* DSN is introduced to extract edge feature information from the original image and fuse the information back with the original image to enhance the boundary features of the original image, hence improving detection accuracy in a congested environment with dense targets overlap.
2. *Introduction of Context-based Feature Fusion Module (CFFB)* CFFB is introduced to realize the fusion of cross-scale feature information, enhance the expression ability of deep networks with high receptive fields, and eventually improve the detection performance on small objects.
3. *SIOU loss function* With the loss function instead of the original loss function, The loss function calculation method is optimized by replacing CIOU with SIOU. In SIOU, the angle deviation factor between the real box and the prediction box is comprehensively considered to improve the prediction confidence and model robustness.

Experimental results show that the DCYOLO algorithm proposed in this paper can effectively improve the accuracy of object detection.

## Related work

### Deep learning object detection algorithm 

Since the introduction of deep learning-based target detection algorithms, many excellent detection algorithms, including the RCNN series and YOLO series, have been produced and continuously improved by scholars all over the world. They have also been used to detect in various scenarios, including the environment with dense objects and urban street object detection.

To better realize the detection of small target objects, YOLOv4 introduced a Path Aggregation Network^8^ (PAN) to further improve the amount of output information, eventually improving the detection performance on small objects, but not the occluded objects. Tan et al.^9^ proposed a BiFPN feature fusion network, which combined the top-down and bottom-up feature extraction processes. BiFPN changed the proportion of each layer’s features by configuring the weights of each layer during the fusion process and extracting sufficient input features through multiple layers. BiFPN improved the detection performance on small objects, but not occluded objects. The EFPN proposed by Deng et al.^10^, that is, the extended feature pyramid network with an additional feature texture migration module, which can extract more small target detail features, thereby improving detection on small targets. But there was no enhancement of the features of the occluded object. Yang et al.^11^ proposed City-YOLO to solve the low accuracy problem of detecting small and dense objects in urban street scenes. City-YOLO was based on YOLOv5 and had an additional detection feature layer with an attention mechanism. Compared with YOLOv5, mAP50 and mAP50:95 of the City-YOLO model were improved by 5.8 and 4.0 percentage points respectively. However, the City-YOLO was less robust because the performance was highly affected by weather.

For better detection performance on dense and occluded targets, Xu Chengji et al.^12^ introduced the attention mechanism based on YOLOV3 and proposed the Attention-YOLO algorithm that improved detection accuracy but lacked accuracy of detection of small targets. Based on YOLOV4, Zhu et al.^13^ replaced the original continuous convolution with the residual connection to generate feature maps of different scales, so the model can obtain a good detection effect in densely distributed environments. However, there was still poor detection performance on small targets. Using RetinaNet as the basic framework, Zhou Dake et al.^14^ proposed a dense human detection algorithm with a dual attention mechanism, which introduced a spatial attention mechanism in the regression process and added a channel attention subnetwork to the classification branch, that effectively improved the detection performance of pedestrians in heavily occluded scenes but with a relatively low detection frame rate of only 11.8 fps. Yang et al.^15^ improved with an additional small target detection branch that had a smaller detection scale. The improved YOLOv5s had a 2.5 percentage points increase in mAP50 and 3.8 in mAP50:95. However, its dataset is a highway dataset with few target types, and if applied to urban streets, the accuracy will be reduced.

A variety of improved object detection algorithms based on YOLO are mostly transmitting more shallow feature information to the deep layer, providing more abundant feature information to the detection head to improve the accuracy of object detection. However, the feature fusion method mentioned above is only a simple splicing of shallow and deep features without considering the fusion of cross-channel features. Those features between each channel are isolated from each other, so there is no way to effectively extract intergroup information from multiple inputs, hence the detection effect of occluded targets and small targets is still not good. Some other studies have introduced the attention mechanism, which is ineffective in improving the detection of small and occluded targets at the same time.

The complexity of such environments makes the classical and currently available detection model unable to meet the requirements, making it necessary to carry out effective object detection research for objects in congested environments.

## Methods

**Figure 1:**
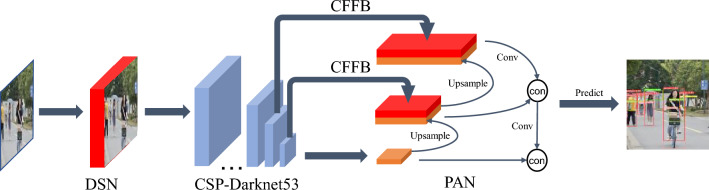
DCYOLO structure.

We propose a new improved model called DCYOLO (Difference Sensitive and Context-based YOLO), which introduces the Difference Sensitive Network (DSN) and the Context-based feature fusion module CFFB based on the YOLOv4 object detection algorithm. Its structure is shown in Fig. 1. The following is a more detailed description of the DSN and CFFB in YOLOv4, DCYOLO, and the loss function.

### YOLOv4

**Figure 2:**
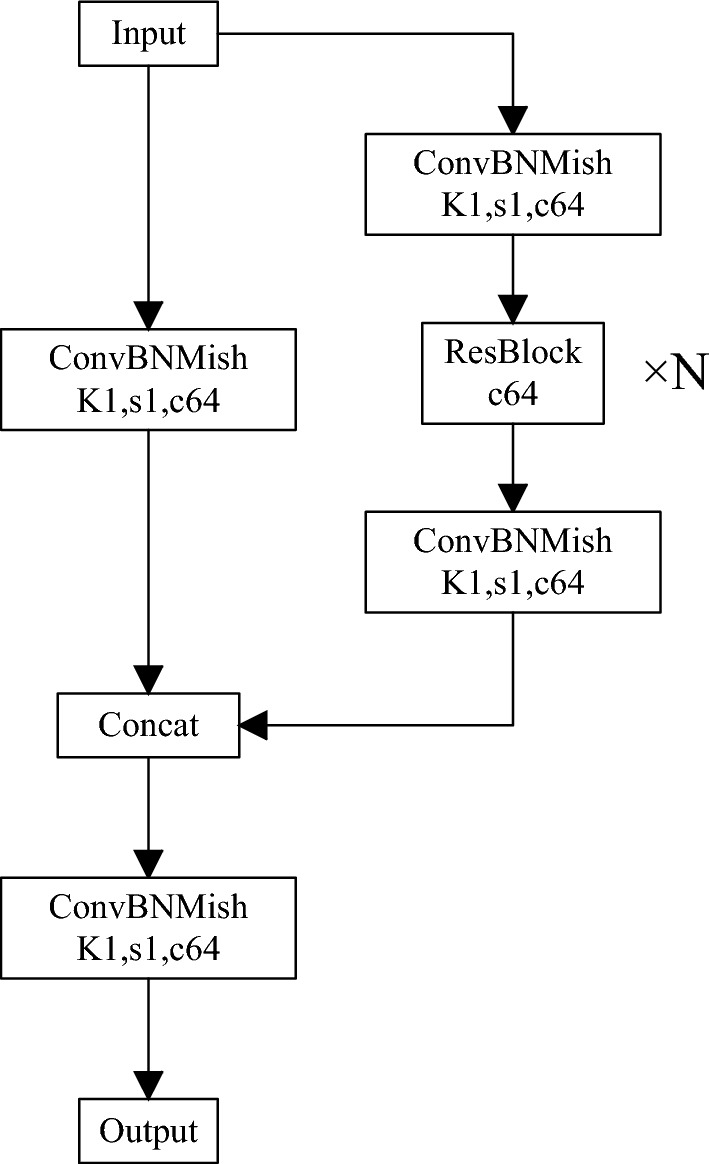
CSPDarknet downsampling.

YOLOv4 uses CSPDarknet53 as the backbone network, and its structure is shown in Fig. 2, obtained after introducing the CSPDensenet module based on Darknet53, which improves the feature fusion capability of shallow and deep networks.

The CSPDarknet consists of six downsampling modules with similar structures described above, and the biggest difference in the structure of each downsampling module is the number of residual blocks. As the network depth increases, the size of the feature map output by each downsampling module gradually decreases, and the semantic level gradually increases. The input with a original size of 416 × 416 × 3 is the output layer with sizes of 208 × 208 × 64, 104 × 104 × 128, 52 × 52 × 256, 26 × 26 × 512, and 13 × 13 × 1024.

**Figure 3:**
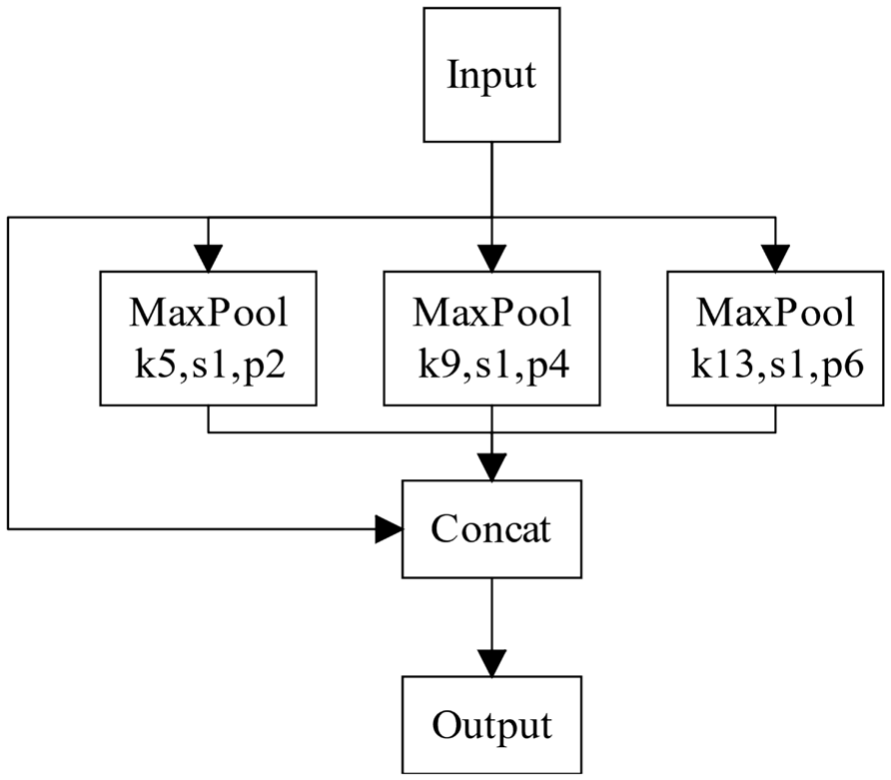
SPP layer for YOLOv4.

The feature map outputted by YOLOv4's backbone network passes through the SPP layer shown in Fig. 3 to obtain a new output feature map with dimensions of 13 × 13 × 2048. The pooling layer of YOLOv4 introduces the original input feature map, which retains a certain amount of original information that is conducive to improving the detection accuracy.

The PAN used by YOLOv4’s neck network is based on the FPN network. At first, PAN performs a top–down upsample, then a bottom–up downsample, and then stitches the feature maps of the same size during the two sampling processes. Unlike the original PAN, YOLOv4 uses channel-oriented stitching instead of the original direct summation, reducing the loss of raw information in shallow networks.

### DCYOLO

DCYOLO consists of four parts, (1) Preprocess: Difference Sensitive Network (DSN); (2) Backbone:CSPDarknet53; (3) Neck: SPP, Context-based Feature Fusion Block (CFFB), PAN; (4) Head:YOLOv4。

**Figure 4:**
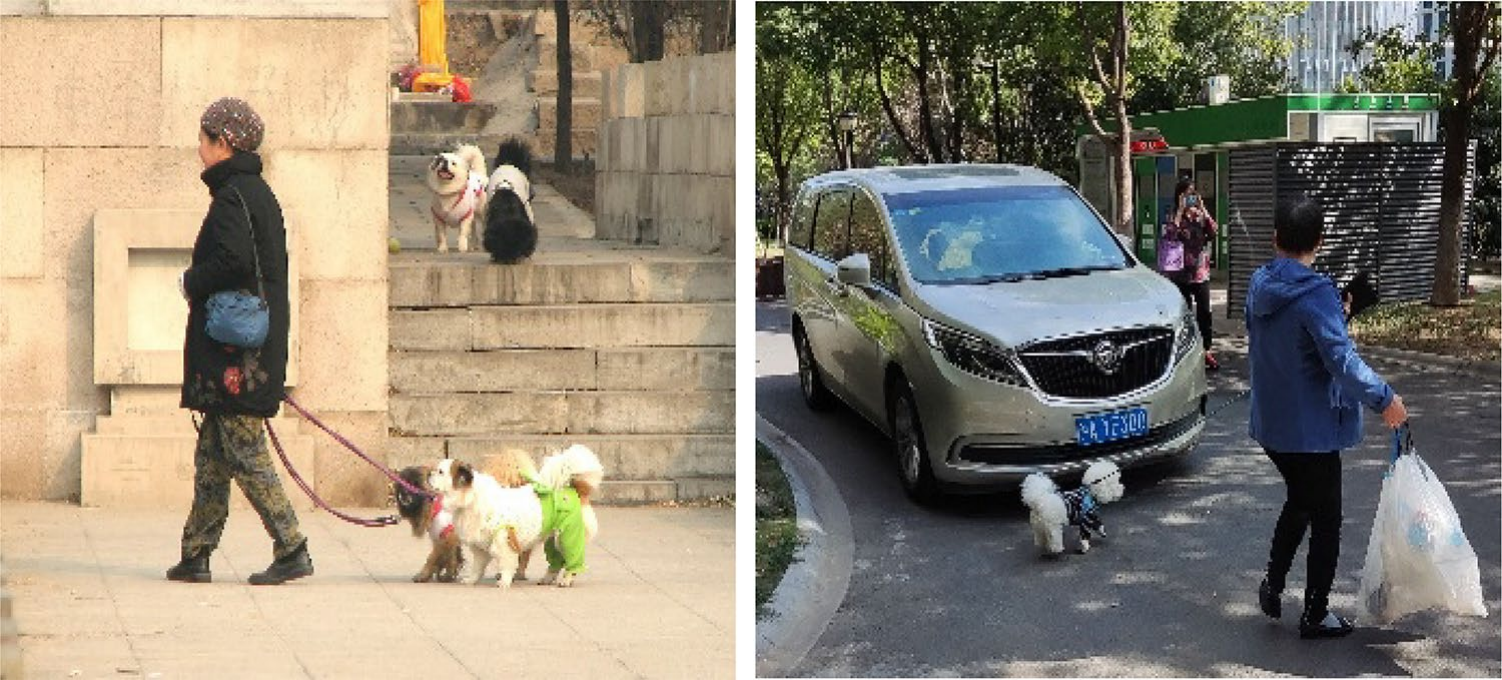
Actual scene diagram.

In many practical scenarios as shown in Fig. 4, there are multiple targets to be detected, with different target scales and overlap. Given this problem, this paper proposes that DSN is used to enhance the image features input into the backbone network and improve both the feature extraction effect and the accuracy of overlapping object detection. Moreover, CFFB is introduced into the PAN structure, and the detailed features extracted from the shallow network are enhanced by the optimized SE^16^ module. Those enhanced features are then fused with the deep features to enhance the fusion of shallow and deep feature information, to improve the detection of occluded and small-sized targets.

#### Difference sensitive network 

In the image, there will be a noticeable difference between the foreground and the background, which can be described to some extent by the intensity of the edges, that is, the gradient. In this process, we consider gradients in both directions at the same time and introduce their corresponding neighborhood information to investigate the changing trend. Those gradients obtained by the fusion can be further summarized and abstracted by the above characteristics, while cross-channel fusion is performed by using the convolution operation with size 1 to enhance the semantic information.

YOLO series detection algorithm directly inputs the original size picture into the backbone network for feature extraction, so that when there are noise, blur, or other influencing factors in the original picture, the desired features will be masked, leading to an unsatisfied object detection effect.

**Figure 5:**
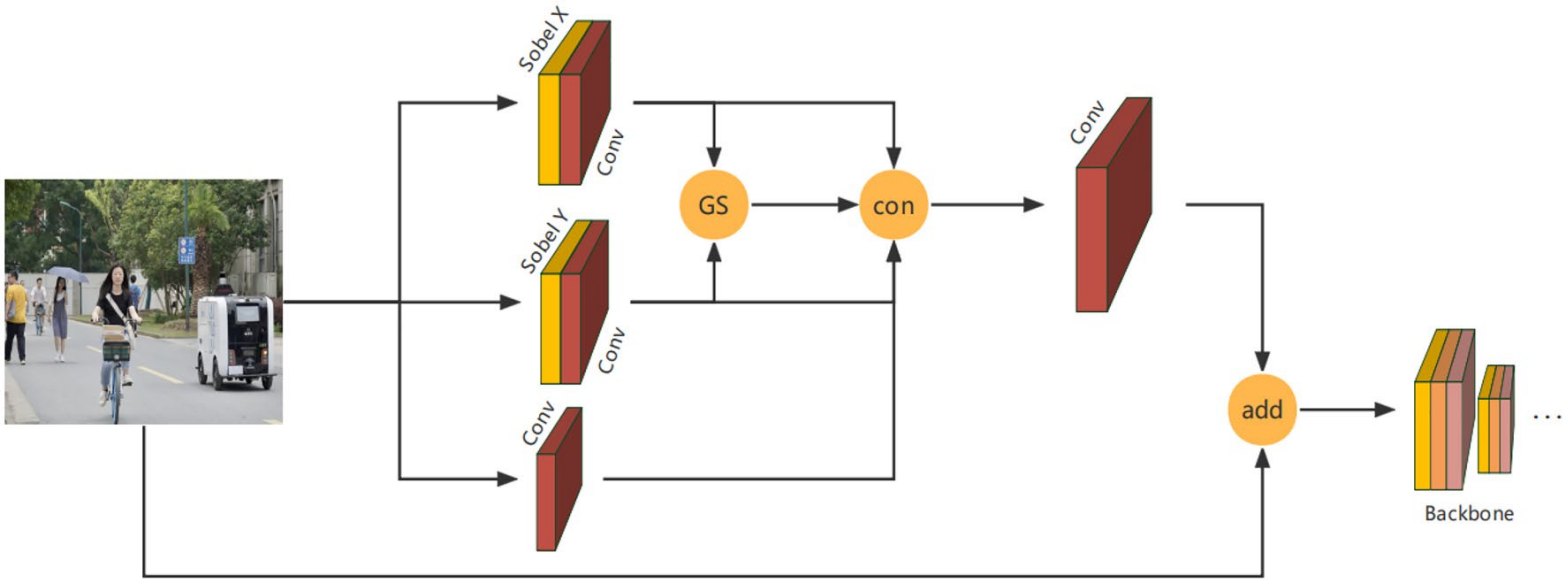
DSN structure.

To deal with the above problems, we add DSN in the YOLOv4 network, the structure of which is shown in Fig. 5. DSN extracts the edge features in the original image in advance, then fuses them with the original image, and then inputs the fusion results into the backbone network. DSN uses the Sobel X and Sobel Y operators to extract the horizontal and vertical gradient features of the original image respectively, realizes cross-channel fusion through convolution kernels, and outputs horizontal gradient feature maps and vertical gradient feature maps respectively with dimensions of 416 × 416 × 1. According to the gradient intensity formula:

1 $$GS=\sqrt{{(dx)}^{2}+{(dy)}^{2}}$$

The gradient value GS on each pixel is computed to obtain the gradient intensity characteristic map, where dx and dy represent the gradient values in the horizontal and vertical directions. The difference-sensitive network completes the separate extraction and synthesis of the gradients in the two directions in the image and describes the feature information of the image by the 416 × 416 × 3 gradient synthesis feature map obtained by GS, dx, and dy stitching, which both retains the original horizontal and vertical feature information and avoids the problem of losing feature information due to the separate description of every single channel. The original image was stitched and convolved with 416 × 416 × C2 channel fusion feature map and gradient comprehensive feature map obtained by 1 × 1 convolution to obtain a global feature map of 416 × 416 × 3. This global feature map obtained through the difference-sensitive network is more sensitive to the target edge information, though it will cause the loss of the original map detail information. To reduce this loss, the original map information will also be directly introduced into the global feature map at the same time to obtain a final output feature map.

Difference-sensitive network makes the model more sensitive to target edge information at the cost of losing a very small amount of detailed information, thereby significantly improving the detection rate of targets with more serious occlusion.

#### Context-based feature fusion block 

The native YOLOv4 detection algorithm extracts the features of the target through the CSPDarknet-53 network, and after the deep convolutional network, the network receptive field is greatly improved, but at the cost of losing certain information, especially of those shallow fine-grained features that play a great role in detecting small targets. The PAN structure extracts the shallow fine-grained features and splices them into deeper networks, to improve the ability of the model to identify targets. Although YOLOv4 introduces the PAN structure, it only superimposes the shallow features directly into the deep feature layer of the same scale without considering the fusion of cross-scale features.

**Figure 6:**
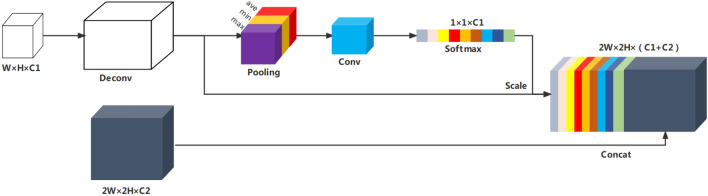
CFFB structure.

**Figure 7:**
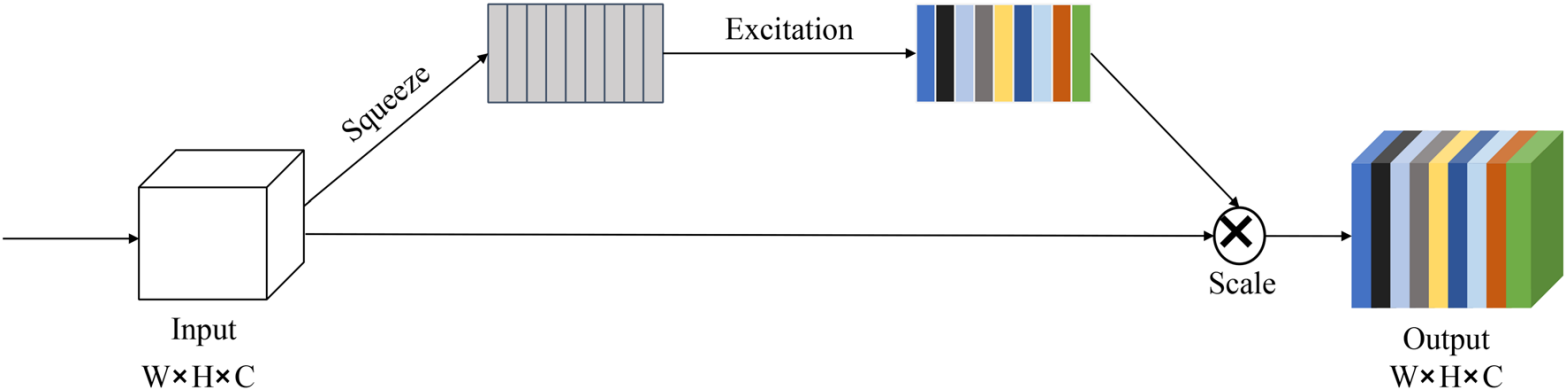
SE mechanism structure.

Given this shortcoming, we propose a feature fusion module (CFFB) based on context information, the specific structure of which is shown in Fig. 6:

CFFB integrates the optimized SE (Squeeze and Excitation) mechanism, which is essentially a model for classifying the characteristics of each channel, and its structure is shown in Fig. 7. The more abstract the features and the higher the semantic level, the more obvious the SE mechanism can make the global features. The pooling layer of the original SE structure adopts global mean pooling, which only uses the average value to describe the characteristics of the entire channel, so more local information is lost during the process. The optimized SE structure uses three non-local statistics, which are the global maximum, global minimum, and global average, to obtain three corresponding feature maps of 1 × 1 × C for the features with input sizes w × h × c. The three maps are then spliced together to obtain a new feature map with a scale of 1 × 1 and a channel number of 3C. Three different pooling layers allow more original context information to be preserved. Subsequent 1 × 1 convolution was performed for cross-channel feature fusion, and the correlation and inhibition between different channels were considered using softmax classification. The cross-scale fusion network first deconvolves the shallow features in the backbone network, and the obtained features are spliced with the feature layer of the same scale in PAN after optimization. The final output features are hence obtained as input for subsequent structures, realizing cross-scale feature fusion.

CFFB can better select features and integrate information of different scales with the help of SE's attention mechanism, so that the features between each channel are no longer isolated. The deep network can then extract effective between-group information from multiple inputs of different scales, thereby improving the detection performance of small targets.

#### SIoU loss function

**Figure 8:**
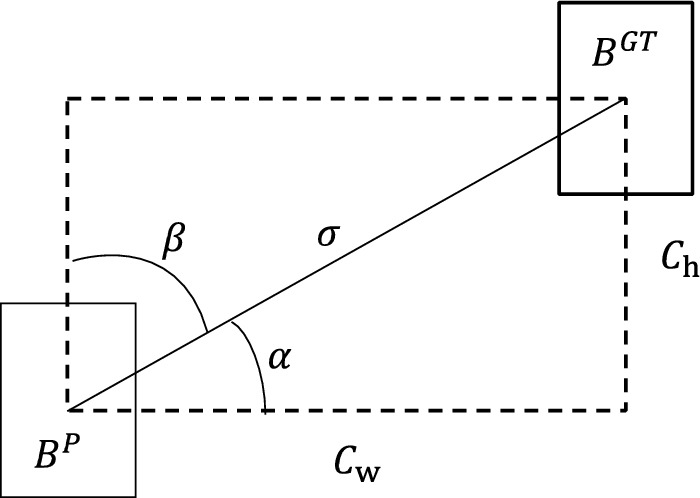
SIoU schematic.

In the original YOLOv4 algorithm, the regression loss of the predicted bounding box is calculated using the CIOU loss function which only considers the loss at the scale level, meaning it does not consider the loss of the prediction box and the real box at the angle. This mismatch of angles can lead to low efficiency and unstable convergence processes, ultimately resulting in poor model accuracy. In this paper, the SCYLLA-IoU (SIoU)^17^ loss function is used instead of the CIoU loss function, which contains four factors: angle, distance, shape, and overlapping area. This is different from the CIoU function, which includes center point distance and aspect ratio instead of overlapping area. A schematic representation of the SIoU loss function is shown in Fig. 8.

In the figure, the ${C}_{w}$ and ${C}_{h}$ are the lateral distance and height difference of the center points of the prediction box ${B}^{P}$ and the real box ${B}^{GT}$, respectively. $\sigma$ is the actual distance of the center point between the two boxes. The angle loss calculation formula is then as follows:

2 $$\Lambda =1-2*{{\text{sin}}}^{2}({\text{arcsin}}\left(\frac{{C}_{h}}{\sigma }\right)-\frac{\pi }{4})$$

Based on the angular loss, recalculate the distance loss:

3 $$\Delta ={\sum }_{t=x,y}(1-{e}^{-\gamma {\rho }_{t}})$$

4 $$\rho_{x} = \left( {\frac{{b_{cx}^{gt} - b_{cx} }}{{C_{w} }}} \right)^{2} ,\;\rho_{y} = \left( {\frac{{b_{cy}^{gt} - b_{cy} }}{{C_{h} }}} \right)^{2} ,\;\gamma = 2 - {\Lambda }$$

When the declination angle $\alpha$ is close to 0, ${C}_{h}$ tends to 0. In such a case, its contribution to the distance loss will be greatly reduced. When $\alpha$ is close to 45°, the contribution of the distance loss is increasing.

Shape loss is defined as follows:

5 $$\mathrm{\varnothing }=\sum_{t=w,h}{(1-{e}^{-{w}_{t}})}^{\theta }$$

Hence, we have

6 $$w_{w} = \frac{{\left| {w - w^{gt} } \right|}}{{{\text{max}}\left( {w,w^{gt} } \right)}},\;w_{h} = \frac{{\left| {h - h^{gt} } \right|}}{{{\text{max}}\left( {h,h^{gt} } \right)}}$$

The equations $(w,h)$ and $({w}^{gt},{h}^{gt})$ are the width and height of the predicted and real boxes. The shape loss can be controlled by adjusting the parameter $\theta$ of which the parameter range is defined in^2,6^.

IoU loss is defined as:

7 $${\text{IoU}}=\frac{{B}^{P}\cap {B}^{GT}}{{B}^{P}\cup {B}^{GT}}$$

According to the above definition of the four loss quantities, the SIoU loss function is defined as follows:

8 $${\text{SIoU}}=1-{\text{IoU}}+\frac{\Delta +\mathrm{\varnothing }}{2}$$

By replacing the CIoU loss function with the SIoU loss function in the original YOLO, the convergence efficiency and the detection accuracy of the model can be improved.

### Informed consent

In addition to the MS COCO dataset, the manuscript includes information/images of participants. We communicated with each participant before capturing their information or images, explained the purpose of photographing, and obtained their consent before including the photos in the manuscript.

For the reasons mentioned above, we can confirm that all participants and/or their legal guardians provided informed consent for the publication of their identifying information/images in open-access online publications.

## Experiments

### Datasets

**Figure 9:**
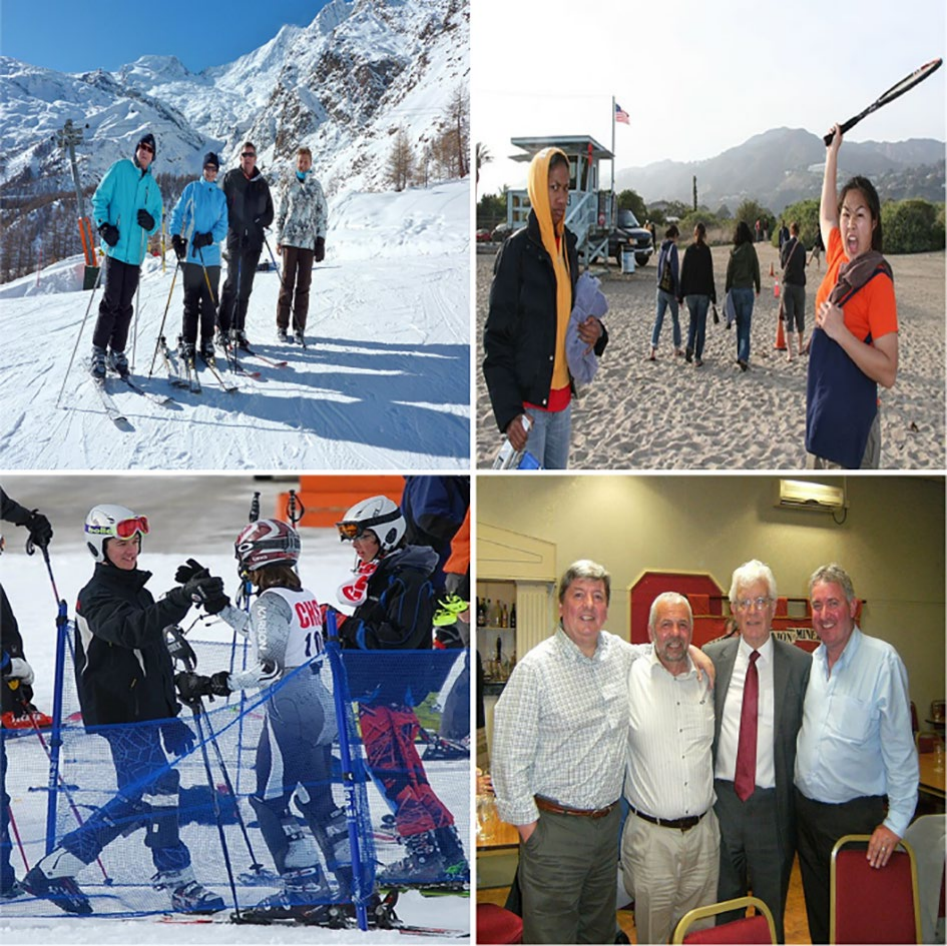
COCO datasets.

In this paper, MS COCO 2017 dataset and self-made dataset are used for model training and evaluation. MS COCO stands for Microsoft Common Objects in Context, which is mainly used to solve three types of problems: object detection, semantic segmentation, and title generation. The COCO dataset contains 2.2 million labeled images, some of which are shown in Fig. 9. The object detection dataset contains 80 categories, including many small targets, which are suitable for the application scenarios mentioned in this article.

**Figure 10:**
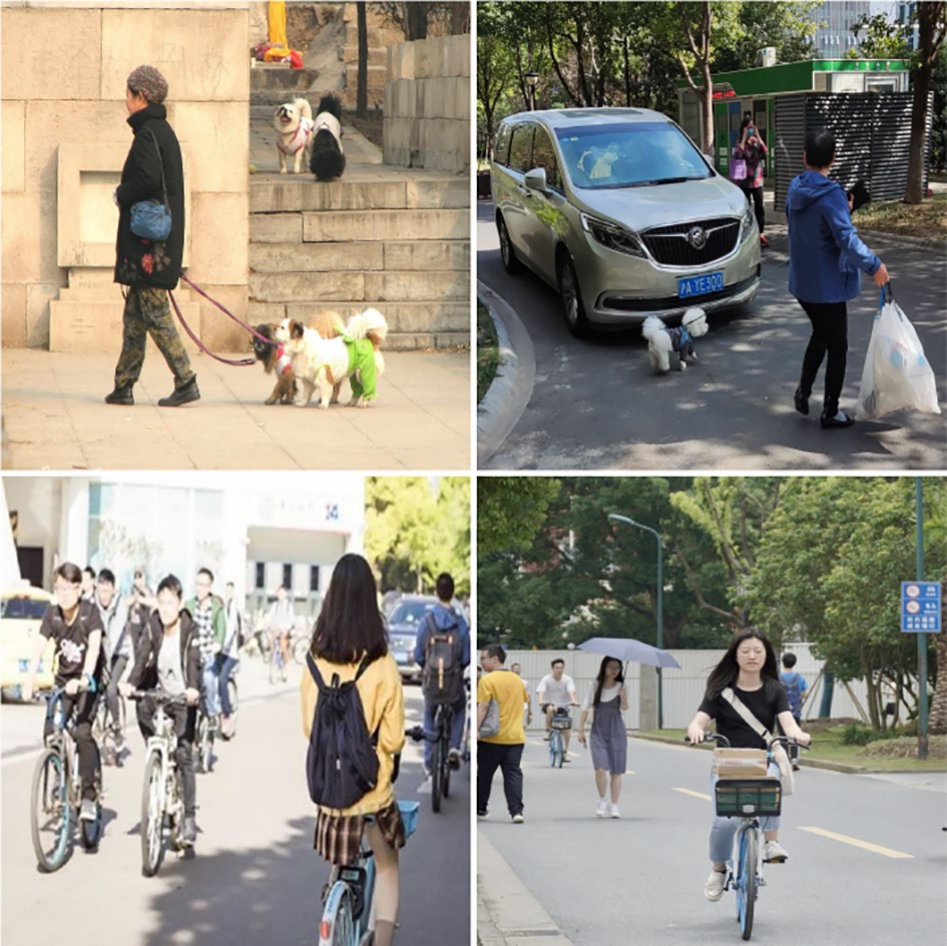
Homemade datasets.

The self-made dataset includes 2000 labeled images, some of which are shown in Fig. 10, in which the target types are humans, cats, dogs, cars, bicycles, motorcycles, etc. The ratio of the training set to the test set is 7:3. In this scenario, the occlusion condition is more serious and has a larger size difference among targets, making it easy for some targets to be missed.

### Training settings

#### Hardware configuration

The model used in this article is based on the Torch framework, implemented using the Python programming language. The system version is Ubuntu 22.04.1. The graphics card is NVIDIA 3070Ti 8G. The CPU model is Intel Core i7-12700H.

#### Training strategies

In this paper, the experiment is first trained on COCO and its own data training set. The model is then evaluated on the test set. In the training stage, the weights obtained from the pre-training of the ImageNet dataset are first used to initialize the CSPDarkNet53 backbone network. The experimental environment is set according to the native YOLOv4 network, and the weight update method is selected by stochastic gradient descent (SGD). The initial learning rate is set to 0.01, and the total number of iterations is 90 k. According to the increase in the number of iterations, the learning rate is reduced to one-tenth at 50 k and 80 k, respectively. The final learning rate reaches 0.001.

#### Data enhancement

**Figure 11:**
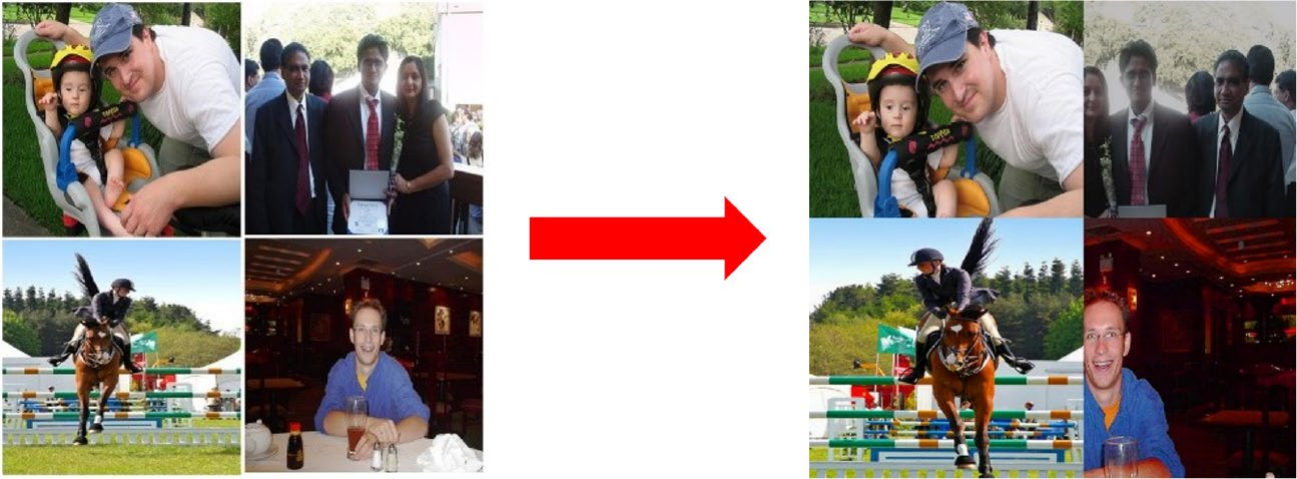
Mosaic processing effect.

Before training, the Mosaic data enhancement method is used to flip, zoom, color gamut change and other operations on four pictures each time. They are then combined into one picture to enrich the background of the detected object. The processing effect is shown in Fig. 11.

### Evaluation indicators

In the process of object detection, IoU is often used to measure the degree of coincidence between the prediction box and the real box, to determine the positive and negative samples. Its calculation formula is as follows:

9 $$IOU=\frac{A\cap B}{A\cup B}$$

When the IoU value of the prediction box and the real box is greater than the set threshold, the prediction is judged to be positive and negative. In order to verify the effect of the model, the following evaluation indexes are used: average accuracy ($AP$) when the IoU threshold is 0.5:0.05:0.95, detection accuracy ($A{P}_{s}$) of small targets ($area<{32}^{2}$), detection accuracy of medium target (${32}^{2}<area<{96}^{2}$), detection accuracy ($A{P}_{m}$) of large targets ($area>{96}^{2}$), and detection accuracy ($A{P}_{l}$).

### Experimental results

#### Experiment and analysis of DSN 

The difference-sensitive network first considers the gradients dx and dy in the two directions in the image, and then calculates and evaluates the influence of the introduction of the comprehensive intensity GS and the 1 × 1 convolution kernel on the model. Therefore, this experiment is performed by introducing different submodules.

As shown in Table 1, each submodule is introduced in the experiment, and the enhancement effect of the difference-sensitive network is verified according to the experimental results obtained in sequence. The experimental results show that when the $1\times 1$ convolution kernel was introduced separately, the model verification accuracy was almost unchanged. When the directional gradient $dx$ and $dy$ were introduced separately, the $AP$ value increased by 0.3 and 0.2 percentage points, the $A{P}_{s}$ increased by 1.6 and 1.4 percentage points, and the $A{P}_{m}$ increased by 0.9 and 1 percentage point, respectively. After the introduction of $GS$, $AP$ increased by 0.4 percentage points, and $A{P}_{s}$, $A{P}_{m}$, and $A{P}_{l}$ increased by 1.1, 0.6, and 0.2 percentage points, respectively. When $dx$,$dy$, and $GS$ were comprehensively introduced, $AP$ increased significantly by 1.8 percentage points, $A{P}_{s}$, $A{P}_{m}$, and $A{P}_{l}$ increased by 3.5, 1.9, and 1.0 percentage points respectively. After introducing the 1 × 1 convolution kernel, the $AP$ value of the model reached the optimal 44.1%. Therefore, for the object detection model, the difference-sensitive network introduced in this paper can significantly improve the detection accuracy of the object detection model.

**Table 1:** Comparison of the effects of DSN submodules.

$dx$	$dy$	$GS$	Conv 1xl	AP	$A{P}_{s}$	$A{P}_{m}$	$A{P}_{l}$
				42.1	21.5	44.8	57.1
✓				42.4	23.1	45.7	57.4
	✓			42.3	22.9	45.8	57.5
		✓		42.5	22.6	45.4	57.3
			✓	42.1	21.6	44.8	57.1
✓	✓			43.1	24.5	45.9	57.7
✓	✓	✓		**43.9**	**25.0**	**46.7**	**58.1**
✓	✓	✓	✓	**44.1**	**25.3**	**46.9**	**58.2**

Significant values are in bold.

**Figure 12:**
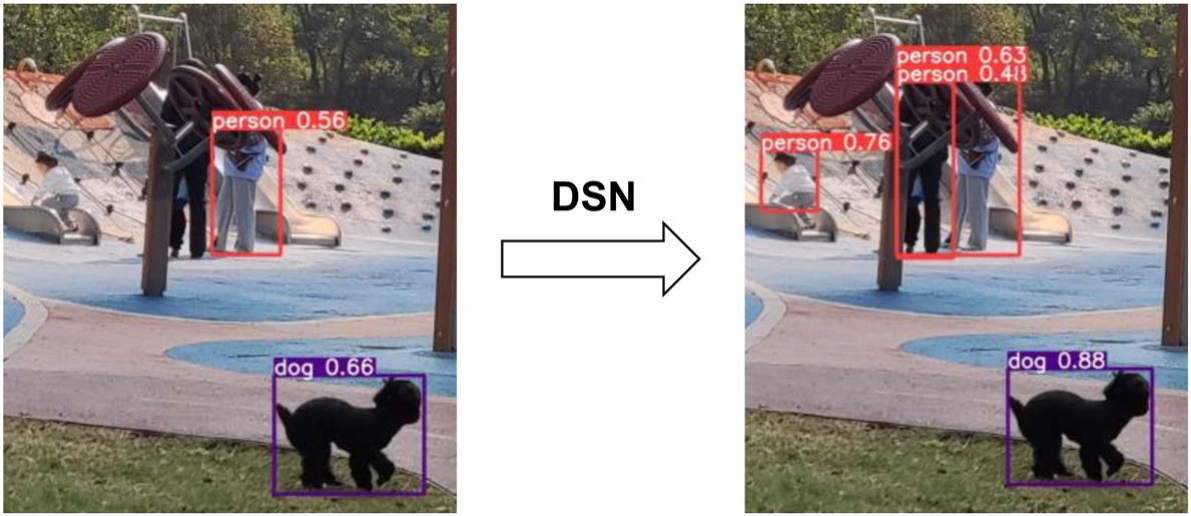
Rendering of DSN's improvement on occluded object detection.

As shown in Fig. 12, in the actual detection process, the network improves the detection accuracy of the original YOLOV4 and greatly improves the detection effect of the occluded objects.

#### Experiment and analysis of CFFB

The feature fusion network needs to use two feature maps of different dimensions as input at the same time. The native YOLOv4 model has three-dimensional feature maps, which are $13\times 13, 26\times 26, and 52\times 52$. That means there are three cross-scale feature combinations. Therefore, the three-dimensional feature map fusion methods are compared and experimented with, and the results are shown in Table 2.

**Table 2:** Comparison of the effects of three CFFB fusion methods.

$13\&26$	$26\&52$	$AP$	$A{P}_{s}$	$A{P}_{m}$	$A{P}_{l}$
		42.1	21.5	44.8	57.1
✓		46.8	25.6	46.1	57.5
	✓	46.3	24.9	45.4	57.3
✓	✓	**48.5**	**28.7**	**46.6**	**57.6**

Significant values are in bold.

According to the results of the table experiment, when the cross-scale feature fusion module was used to fuse $13\times 13$ and $26\times 26$ or $52\times 52$ and $26\times 26$ separately, $AP$ increased by 0.7 and 0.9 percentage points, respectively. When the two cross-scale fusion modules were introduced at the same time, the model accuracy reached the best, and the $AP$ value increased by 1.4 percentage points. Therefore, the context-based cross-scale feature fusion network proposed in this paper can significantly improve the detection accuracy of the object detection model.

**Figure 13:**
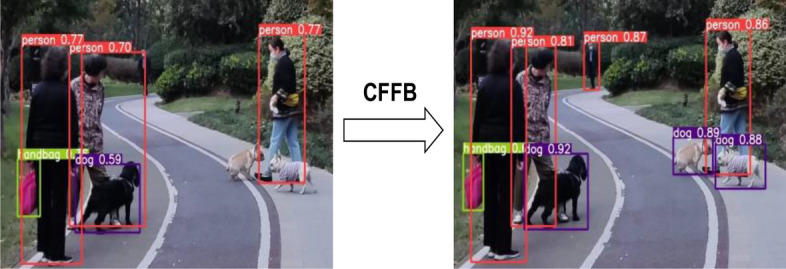
Rendering of CFFB's improvement in small-size object detection.

As shown in Fig. 13, in the actual detection process, the network not only improves the detection accuracy of the original YOLOV4, but also greatly improves the detection effect of small-sized targets.

#### Experiment and analysis of loss function

To better verify the improvement effect, this section compares the SIoU loss function with both the CIoU loss function and the commonly used GioU and DioU loss functions in the original YOLOv4 model. The experimental results are shown in Table 3.

**Table 3:** Comparison of the effects of different loss function algorithms.

Model	Loss	$AP$	$A{P}_{S}$	$A{P}_{m}$	$A{P}_{l}$
DCYOLO	SIoU	**51.2**	**31.9**	**49.0**	**59.3**
DCYOLO	CIoU	49.8	30.1	48.3	58.8
DCYOLO	DIoU	49.4	29.2	47.9	58.6
DCYOLO	GIoU	48.8	28.4	47.2	58.4

Significant values are in bold.

The experimental results show that the accuracy of the model trained using the SIoU loss function is the highest, and the $AP$ value of the obtained model is 2.4, 1.8, and 1.4 percentage points higher than that of GIoU^18^, DIoU, and CIoU^19^, respectively. Therefore, the accuracy of the detection model trained using the SIoU loss function is better than those of other loss functions, which is more suitable for the DCYOLO model proposed in this paper.

#### Comparison of model detection effects

To verify the performance of detection, DCYOLO was compared with the more advanced object detection algorithms in this paper on the test dataset and the result is shown in Table 4.

**Table 4:** Comparison of the effects of different detection algorithms.

Model	DSN	CFFB	$AP$	$A{P}_{S}$	$A{P}_{m}$	$A{P}_{l}$
Faster-R-CNN	×	×	35.0	16.2	39.2	50.8
Faster R-CNN w FPN	×	×	36.5	18.9	38.3	47.9
YOLOv3	×	×	33.5	19.1	35.8	41.9
YOLOv4	×	×	42.1	21.5	44.8	57.1
YOLOV5x	×	×	47.6	29.1	45.8	58.4
YOLOv4	√	×	**44.1**	25.3	46.9	58.2
YOLOv4	×	√	**48.5**	28.7	46.6	57.6
**DCYOLO**	√	√	**51.2**	**31.9**	**49.0**	**59.3**

Significant values are in bold.

The experimental results showed that the DCYOLO achieved the highest detection accuracy after integrating DSN and CFFB. Compared with YOLOv4, the $AP$ of DCYOLO increased by 9.1 percentage points, which is already higher than those of other detection algorithms. ${AP}_{s}$, ${AP}_{m}$, and ${AP}_{l}$ of DCYOLO increased by 10.4, 4.2, and 2.2 percentage points, respectively. The result showed that the detection accuracy of DCYOLO on various types of objects, especially small and medium-sized targets, was significantly improved.

Compared with the Faster RCNN-related object detection algorithms, DCYOLO's $AP$ value was higher. Therefore, compared with other algorithms, DCYOLO has a prospective advantage.

**Figure 14:**
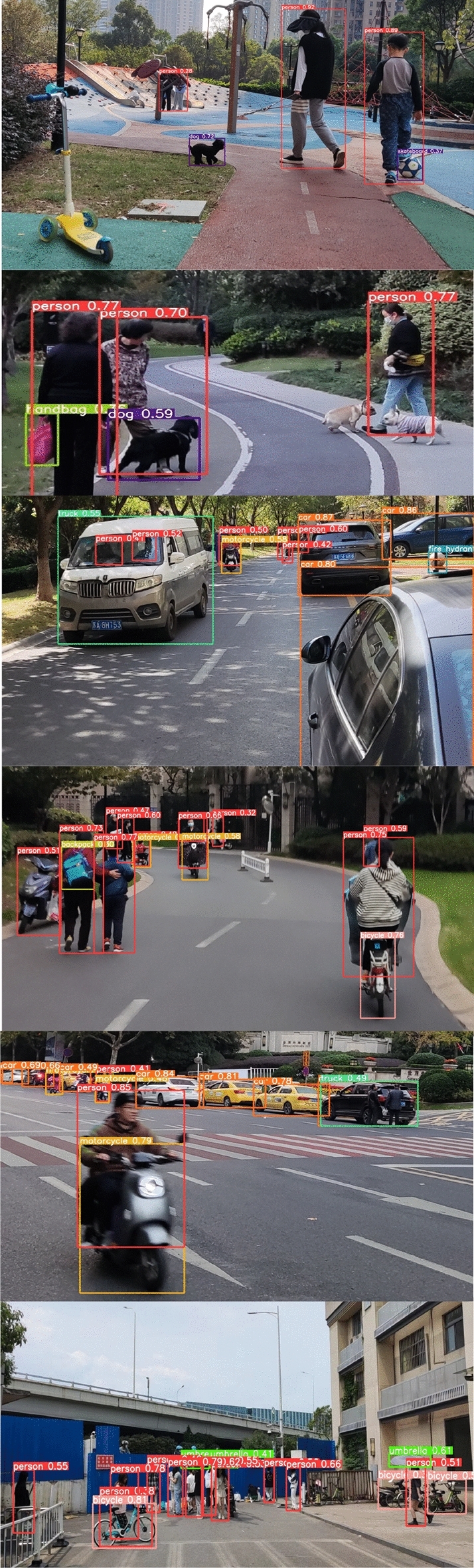
YOLOV5x test results.

**Figure 15:**
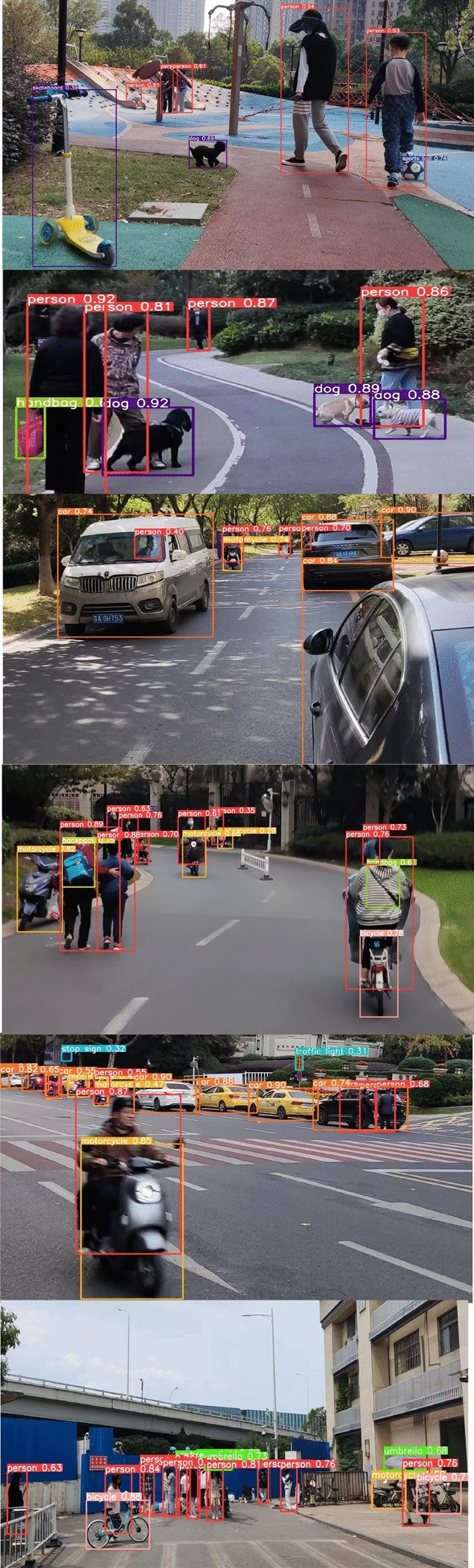
DCYOLO test results.

Figures 14 and 15 show the comparison between the DCYOLO algorithm and the current advanced YOLOv5x algorithm detection effect, respectively, and it can be seen that the DCYOLO algorithm can better detect overlapping targets and small size targets that are heavily occluded, and have better detection effects.

## Conclusion

To improve the performance of object detection in the congested environment of urban streets, this paper proposes a new improved object detection algorithm of DCYOLO. DCYOLO introduces the difference-sensitive network (DSN) and the cross-scale feature fusion module (CFFB) based on context information. The loss function is also improved, leading to a higher accuracy of the algorithm. The experimental results show and prove that: DCYOLO performs better in dense object environments than other current object detection algorithms. From the experimental results, we observe and conclude the following points:

- With the addition of DSN, AP and AP_s_ of the original model respectively gained an increase of 2 and 3.8 percentage points. We conclude that DSN can extract more contour information from the object in the images, thereby improving the detection performance in dense environments with size-differentiated and occluded objects.
- With the addition of CFFB, AP and AP_s_ of the original model respectively gained an increase of 6.4 and 7.2 percentage points. We conclude that CFFB introduces fine-grained features of shallow networks into deep networks, thereby improving the detection performance, especially for small targets.
- The SIoU loss function takes the angular deviation factor between the real box and the predicted box into account, thereby improving the accuracy of the model.
- The complete DCYOLO brought significant overall improvement in detection performance for the original model, with an increase of 9.1, 10.4, 4.2, and 2.2 percentage points for AP, AP_s_, AP_m_, and AP_l_, respectively.

Compared with the original YOLOv4, YOLOv5x, and Faster R-CNN models, DCYOLO has better detection performance with higher detection accuracy. Nevertheless, in the experiments, there are still some small-sized and occluded objects that cannot be detected accurately. The continuous optimization of DCYOLO will be one of the main tasks in our future research. In addition, we realize that although DCYOLO is derived from YOLOv4, it will not be limited to YOLOv4. DCYOLO provides a new idea for the optimization of the object detection model. We believe that the idea proposed in this paper will be used in newer and more advanced models in the future to adapt to more complex environments, which is another major task in future studies.

## Data Availability

The datasets generated and/or analyzed during the current study are available in the MS COCO dataset which is a public dataset that is available from a given URL for everyone according to their needs (https://cocodataset.org/#home).
